# Pathogen spectrum of community acquired pneumonia in people living with HIV (PLWH) in the German CAPNETZ-Cohort

**DOI:** 10.1007/s15010-023-02070-3

**Published:** 2023-07-09

**Authors:** Benjamin T. Schleenvoigt, Juliane Ankert, Grit Barten-Neiner, Florian Voit, Norbert Suttorp, Christoph Boesecke, Christian Hoffmann, Daiana Stolz, Mathias W. Pletz, Gernot Rohde, Martin Witzenrath, Marcus Panning, Andreas Essig, Jan Rupp, Olaf Degen, Christoph Stephan, for the CAPNETZ Study Group, M. Dreher, C. Cornelissen, W. Knüppel, P. Creutz, A. Mikolajewska, A. le Claire, M. Benzke, T. Bauer, D. Krieger, M. Prediger, S. Schmager, M. Kolditz, B. Schulte-Hubbert, S. Langner, A. Hüfner, T. Welte, J. Freise, M. Nawrocki, I. Fuge, J. Freise, J. Naim, W. Kröner, T. Illig, N. Klopp, C. Kroegel, A. Moeser, C. Bahrs, D. Drömann, P. Parschke, K. Franzen, N. Käding, M. Wouters, K. Walraven, D. Braeken, H. Buschmann, A. Zaruchas, T. Schaberg, I. Hering, W. Albrich, F. Waldeck, F. Rassouli, S. Baldesberger, M. Wallner

**Affiliations:** 1 Institute of Infectious Diseases and Infection Control, Jena University Hospital/Friedrich-Schiller-University, Jena, Germany; 2 CAPNETZ STIFTUNG, Hannover, Germany; 3 Department of Internal Medicine II, School of Medicine, University Hospital rechts der Isar, Technical University of Munich, Ismaninger Str. 22, 81675 Munich, Germany; 4 Department of Infectious Diseases and Respiratory Medicine, Charité-Universitätsmedizin Berlin, Corporate Member of Freie Universität Berlin and Humboldt-Universität zu Berlin, Charitéplatz 1, 10117 Berlin, Germany; 5 German Center for Lung Research (DZL), Berlin, Germany; 6 Department of Internal Medicine I, University Hospital Bonn, Sigmund-Freud-Str. 25, 53127 Bonn, Germany; 7 ICH Study Center Hamburg, Hamburg, Germany; 8 University Hospital of Schleswig-Holstein, Campus Kiel, Kiel, Germany; 9 Department of Pneumology, University Medical Center Freiburg, Freiburg, Germany; 10 Medical Department I, Department of Respiratory Medicine, Goethe University Hospital, Frankfurt/Main, Germany; 11 Biomedical Research in Endstage and Obstructive Lung Disease Hannover (BREATH), German Center for Lung Research (DZL), Hannover, Germany; 12 Department of Infectious Diseases, Respiratory and Critical Care, Charité-Universitätsmedizin Berlin, Berlin, Germany; 13 Institute of Virology, University Medical Center-University of Freiburg, Freiburg, Germany; 14 Institute of Medical Microbiology and Hygiene, University Hospital of Ulm, Ulm, Germany; 15 Department of Infectious Diseases and Microbiology, University Hospital Schleswig-Holstein, Lübeck, Germany; 16 German Center for Infection Research (DZIF), Partner Site Hamburg-Lübeck-Borstel, Giessen, Germany; 17 Outpatient Infectious Diseases Unit, University Medical Center Hamburg Eppendorf, Hamburg, Germany; 18 Medical Department II, Section Infectious Diseases, University Medical Center, Frankfurt am Main, Germany

**Keywords:** HIV, CAP, PLWH, Pneumonia, Community acquired pneumonia, Empirical antibiotic treatment

## Abstract

**Objectives:**

The objective of this study was to identify the pathogen spectrum of community acquired pneumonia in people living with HIV (PLWH), and to compare it with a matched HIV negative group in order to reassess therapeutic strategies for PLWH.

**Methods:**

Seventy-three (n = 73) PLWH (median CD4 3–6 months before CAP: 515/µl; SD 309) with community acquired pneumonia (CAP) were matched with 218 HIV-negative CAP controls in a prospective study design. Pathogen identifications used blood culture, samples from the upper and lower respiratory tract (culture and multiplex PCR) and urinary pneumococcal and legionella antigen test.

**Results:**

Although the vaccination rate among PLWH with CAP was significantly higher (pneumococcal vaccination: 27.4 vs. 8.3%, p < 0.001; influenza vaccination: 34.2 vs. 17.4%, p = 0.009), pneumococci were found most frequently as pathogen among both PLWH (n = 19/21.3%) and controls (n = 34/17.2%; p = 0.410), followed by *Haemophilus influenzae* (PLWH, n = 12/13.5%, vs. controls, n = 25 / 12.6%; p = 0.850). *Staphylococcus aureus* was found equally in 20.2 and 19.2% in PLWH and controls, but infection or colonization could not be distinguished. Mortality during 6-month follow-up was significantly higher for PLWH (5/73, or 6.8%) versus controls (3/218, or 1.4%), however with lower case numbers than previously reported. Typical HIV-associated pathogens such as *Pneumocystis jirovecii* were found only exceptionally.

**Conclusions:**

Our study underscores the persistent clinical burden of CAP for PLWH. From pathogen perspective, empirical antibiotic treatment for CAP in PLWH on antiretroviral therapy should cover pneumococci and *Haemophilus influenzae* and may be adopted from valid common recommendations*.*

**Supplementary Information:**

The online version contains supplementary material available at 10.1007/s15010-023-02070-3.

## Introduction

For people living with HIV (PLWH), community acquired pneumonia (CAP) is one of the main reasons for hospitalization and recurrent CAP is an AIDS-defining disease [1]. Nevertheless, little is known about the incidence and the spectrum of pathogens in this vulnerable patient group and the available data are not up-to-date and were mainly generated before the HAART (highly active antiretroviral therapy) era. Data from France, appearing in 2010, found in a prospective cohort study a comparably high overall incidence of pneumonia for PLWH as it is also known for HIV negative individuals. However, the distribution of age, gender, immune status and the level of HIV viral load may be significantly different for PLWH with CAP and those without HIV. The incidence for pneumonia within the 30–40-year-old age group in this French cohort was 13.6/1000 for PLWH. Concerning gender distribution, the CAP-incidence among PLWH was higher for women, compared to men (15.1/1000 vs. 10.9 /1000), which is in contrast to the sex distribution of non-HIV-CAP patients [2]. With CD4 values of 200 to 350/µl and increased viral loads of > 1000 copies of HIV-RNA/ml, incidences of 16.9/1000 are given [3]. Based on these data, and thus assuming a CAP incidence of 11 to 15/1000 PLWH in 84.700 HIV-positive people in Germany (2016), we estimated a CAP disease burden of 932–1271 annual cases [4]. A current investigation from the Swiss HIV Cohort Study (SHCS), presented recently at European AIDS Congress (EACS), confirms the incidence estimate of HIV/CAP in Germany from 2017. These new data indicate that recent achievements in the antiretroviral treatment and HIV infection prevalence, with long-term stable suppressed HIV viral loads, high immunological regeneration, in combination with less nicotine abuse, led to a decreasing incidence of CAP in PLWH. From 2008 to 2018, 985 bacterial pneumonia episodes were observed in more than 12.000 patients with HIV infection in SHCS, with a decreasing annual case incidence from around 13/1000 in 2008, to 7/1000 person-years in 2018. Incidence was significantly dependent on factors such as age, level of education, intravenous drug use, nicotine abuse, cellular immune status, HIV viral load and previous pneumonia. Unfortunately, the causative pneumonia pathogens were not investigated in SHCS [1], therefore, it is not known, whether the spectrum of CAP-pathogens in PLWH differs from that in HIV uninfected individuals.

Objective of this work was to identify the pathogen spectrum of CAP in PLWH, and to compare it with a matched HIV negative comparator group, in order to identify differences or similarities in the pathogen spectrum and moreover, to confirm equal therapeutic strategies for PLWH and HIV uninfected patients with CAP.

## Methods

The CAPNETZ study project prospectively documented n = 74 study patients with HIV infection and confirmed CAP from January 31, 2017 to March 9, 2021. Inclusion criteria were: Age ≥ 18 years, imaging pulmonary infiltrate (chest X-ray or CT) and at least one of the following items: cough, purulent sputum, positive clinical auscultation result or fever ≥ 38.3 °C (rectal) or ≥ 37.8 °C (axillary/oral/auricular/sublingual). Excluded from the study were patients with none infectious infiltrates, active pulmonary tuberculosis in the last 2 month and hospitalization more than 48 h prior to the diagnosis of current pneumonia. For HIV infected patients, demographic data, recorded risk factors for CAP and HIV-specific parameters for virological and immunological status and antiretroviral treatment were documented. During the same period, n = 1.358 HIV-negative study patients with CAP were included in the study. This total cohort was examined microbiologically with respect to causative pathogens. A detailed description of the CAPNETZ methodology is given elsewhere [5]. The diagnostic methods and processes may differ between the study sites, because the patients are documented according to the respective clinical routine. However, the laboratories are subject to standardized certification.

One patient out of 74 with underlying immunosuppression other than HIV was excluded in the study group. All 218 controls had no immunosuppressive precondition. Study patients and controls were recruited multicentric in 31 different CAPNETZ sites in Germany. A detailed list of study sites is provided in the Supplement section.

### Statistical analysis

Statistical analysis was performed using SPSS Statistics version 28 and Prism GraphPad 9.0. The data set was first matched 1:3 for age and sex (PLWH vs. HIV-negative control group) using R software. For normally distributed metric data (tested by Kolmogorov–Smirnov test), the independent variable t test was used, and for nonnormally distributed metric data, the Mann–Whitney U test was used. The χ^2^-test (Fisher's exact test) was used for analysis of nominal or ordinary data and results were corrected for multiple testing by Bonferroni correction. A p value of < 0.05 was considered significant.

Changes in median CD4 levels and HIV viral load at different time points were analyzed using mixed models. In the simple linear regression model, known and unknown factors that might influence mortality were assessed. Individual confounding factors with a potential influence that had a p value < 0.05 in the univariate linear regression model were tested for multicollinearity using Kendall-Tau-B correlation analysis and included in the multiple linear regression analysis if there was no correlation (r < 0.5). Figures were created by GraphPad Prism version 9.0 software.

## Results

### Patient’s characteristics

The characteristics of enrolled study patients and controls are shown in Table 1. Significant differences were found for BMI, current smoking status and prior vaccination status. There are no significant differences concerning current or historical pneumonia characteristics or underlying airway disease.

**Table 1:** Characteristics of HIV-positive patients (PLWH) with CAP (n = 73) and matched HIV-negative controls (n = 218)

	PLWH (n = 73)	Controls (n = 218)	p-value
Age in years (range)	52 (39.5–61)	53 (39.8–62)	0.688
Gender, male	64 (87.7%)	192 (88.1%)	> 0.9999
*Vaccination history*			
Influenza < 12 month (n = 71/196)	25 (34.2%)	38 (17.4%)	0.0091
Pneumococci < 5 years (n = 70/194)	20 (27.4%)	18 (8.3%)	0.0002
PSV (n = 19/9) Pneumococcal polysaccharide vaccine	9 (12.3%)	4 (1.8%)	> 0.9999
PCV (n = 19/9) Pneumococcal conjucate vaccine	14 (19.2%)	7 (3.2%)	> 0.9999
Hospital admission	50 (68.5%)	216 (99.1%)	0.0823
ICU admission	4 (5.7%)	7 (3.2%)	0.477
CRB-65 (n = 62/191)			
0	37 (50.7%)	132 (60.6%)	0.2141
1	22 (30.1%)	54 (24.8%)	0.3387
2	3 (4.1%)	5 (2.3%)	0.3812
Earlier pneumonia (n = 72/207)	26 (35.6%)	53 (24.3%)	0.0963
COPD (n = 72/215)	8 (11.0%)	36 (16.5%)	0.3444
BMI (kg/m^2^) (n = 68/209)	22.8 (20.3–25.7)	26.3 (23.5–30.2)	< 0.001
Current smoker (n = 70/201)	37 (52.9%)	66 (32.8%)	0.0041
Most probable route of HIV infection (n = 67/73)			
Men having sex with men (MSM)	38 (52.1%)		
Intravenous drug use (IVD)	9 (12.3%)		
Contaminated blood infection	3 (4.1%)		
Vertical transmission	1 (1.4%)		
Current I.V. drug user (n = 6)	1 (1.4%)		
CDC stadium (n = 63/73)			
A	22 (35%)		
B	11 (17%)		
C	30 (48%)		
On Antiretroviral Therapy (ART)	65/73 (89%)		
ART-change due to CAP	27/73 (37%)		
Time in years since ART therapy was started (n = 57)	10 (3–16)		
Time in years between initial HIV diagnosis and CAP (n = 71)	12 (4–21)		
Still anti-infective prophylaxis (n/N)	16/73 (21.9%)		
Antibiotics/antituberculosis	n = 11		
Antivirals	n = 8		
Still in therapy due to opportunistic infections (n/N)	n = 11/73 (15.1%)		
PcP	n = 9		
Tuberculosis	n = 1		
HSV 1	n = 1		
CD4 cell count result available at CAP time (n = 27/73)			
> 500/µL	9/27 (33.3%)		
350–500/µL	5/27 (18.5%)		
200–349/µL	4/27 (14.8%)		
< 200/µL	9/27 (33.3%)		

**Fig. 1:**
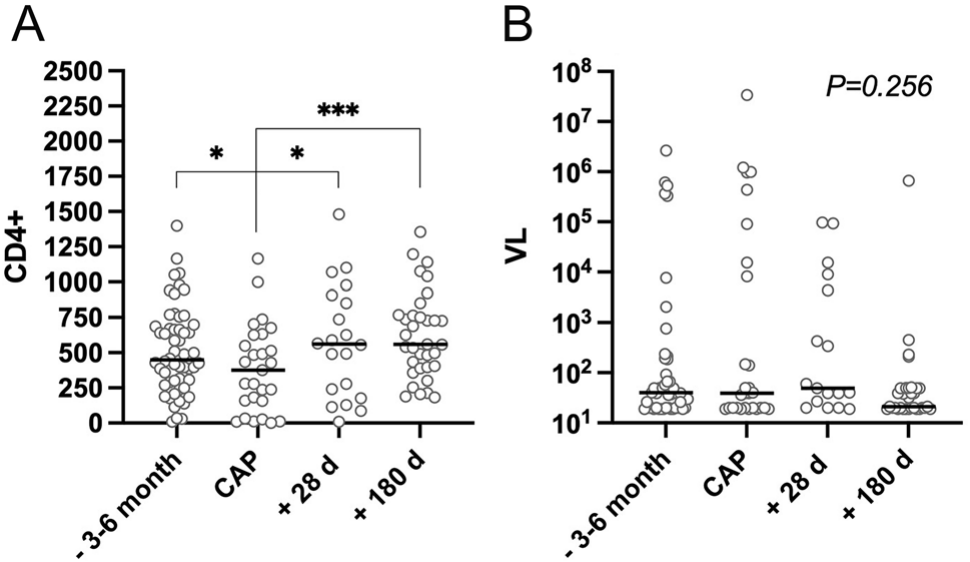
Median and individual values of CD4 (**A**) and viral load (**B**) 3–6 months before, during and 28 and 180 days after CAP. The mixed model was used to determine statistical significance. P-values refer in each case to the time during the CAP and are presented as follows: *p < 0.05, ***p < 0.001

The second part of Table 1 shows the HIV related characteristics at time of CAP for the study patients only (n = 73). The majority of CAP-patients had previously been symptomatic with HIV-associated diseases before, with a strong proportion of patients with full AIDS (> 40%). CD4 cell counts and HIV-RNA before and after CAP are presented in Fig. 1 (see also Supplement Table 1).

Statistically significant differences were observed with regard to vaccination status (Table 1). However, differences between study patients and controls in terms of vaccine type and regimen were not analyzed due to the small numbers of cases in the subgroups.

PLWH with viral load > 50 /ml (n = 12) at CAP were compared with those below (n = 17). Although these subgroups are small, we found a significant difference for current smoking status (HIV viral load > 50/ml: 9/12 (75%) vs. HIV viral load < 50/ml: 8/17 (47%); p = 0.041). The same analysis was conducted for the subgroup with CD4 > / < 200 (n = 18/9). Here we found a significant difference for CDC stage C (CD4 > 200: 5/17 (29.4%) vs. CD4 < 200: 7/8 (87.5%); p = 0.011), BMI (CD4 > 200: 22.8 (21.3–24.9) vs. CD4 < 200: 20.1 (18.8–21.3); p = 0.022) and hospital admission (CD4 > 200: 9/18 (50%) vs. CD4 < 200: 9/9 (100%); p = 0.012). Supplement Table 2 shows the investigated parameters in detail.

### Hospitalization, morbidity and mortality of CAP in PLWH

**Fig. 2:**
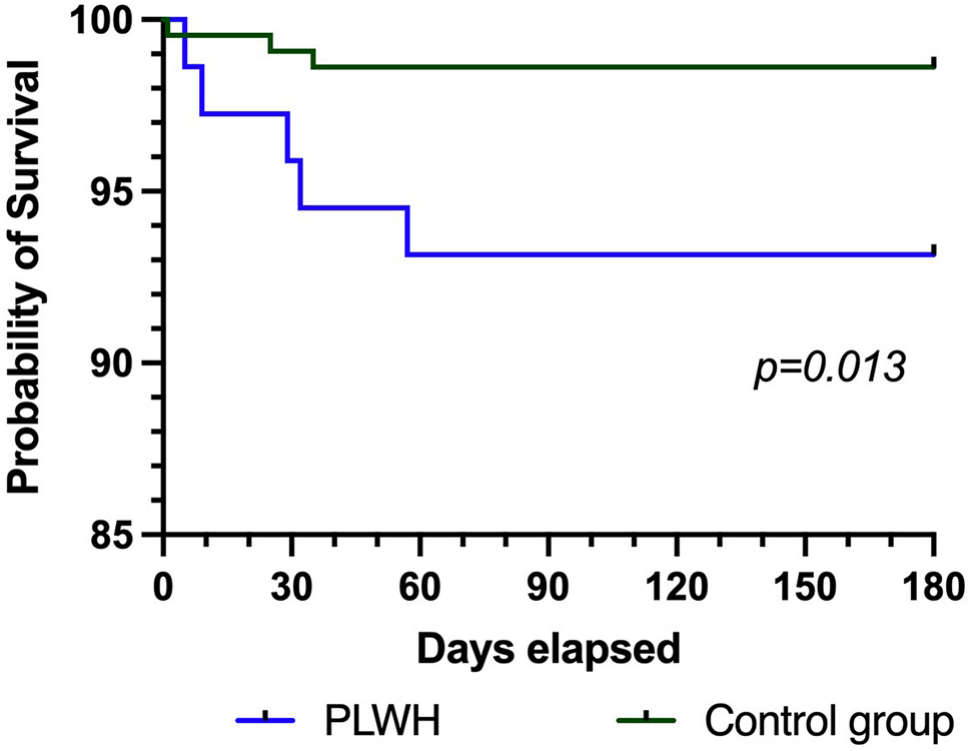
Mortality of HIV-positive patients with CAP and matched HIV-negative controls over six months (Kaplan–Meier curve)*.* Statistical differences between the groups were calculated using the log-rank (Mantel Cox) test. A higher mortality in the PLWH group could be shown (p = 0.013)

Albeit more than 80% of HIV-positive patients with CAP had low CRB-65 Index of only 0 and 1, the hospitalization rate was 68.5% while intensive care treatment was infrequent; no significant differences between PLWH and controls were observed (Table 1). Mortality during 6-month follow-up was higher in PLWH (6.8%) than in control subjects (1.4%) (p = 0.013). These patients died no later than day 60 after CAP diagnosis (Fig. 2).

At least one pathogen was detected in 7 of the deceased patients. Most frequently, in 6 out of the deceased cases, *Staphylococcus aureus* was detected. In 5 of those by PCR from nasopharyngeal swab and by culture. HIV disease stage of all PLWH was C3 (see Supplement Table 3).

In single linear regression analysis, HIV status (p = 0.013), BMI (p = 0.016), and ART use (p = 0.031) were identified as influencing mortality at 180 days. However, none of the three variables could be confirmed in the multiple linear regression analysis. Thus, no clear risk factor on mortality after 180 days could be identified in the present cohort.

### Pathogen detection

Pathogens were detected using antigen detection assays from urine (253 samples), microbiological culture from blood (149), sputum (107) and bronchoalveolar lavage (BAL) (26 samples) and multiplex PCR on viral and bacterial pathogens from nasopharyngeal swab (259), BAL (107) and sputum (26 samples). Laboratory methods were equally distributed among PLWH and controls except culture from BAL, that was significantly more performed in the study group (20.3 vs 4.7%; p < 0.001) (see Supplement Table 8).

At least one pathogen was identified in n = 51 (69.9%) PLWH and in 125 (57.3%) matched controls, without significant difference for probability of detection (p = 0.072). In 31 (42.5%) PLWH- and 58 (26.6%) control-cases, there were more than one pathogen per individual detected, respectively. For 4 and 16 cases, the same pathogens have been detected in one individual by different diagnostic methods for PLWH and controls, respectively.

**Fig. 3:**
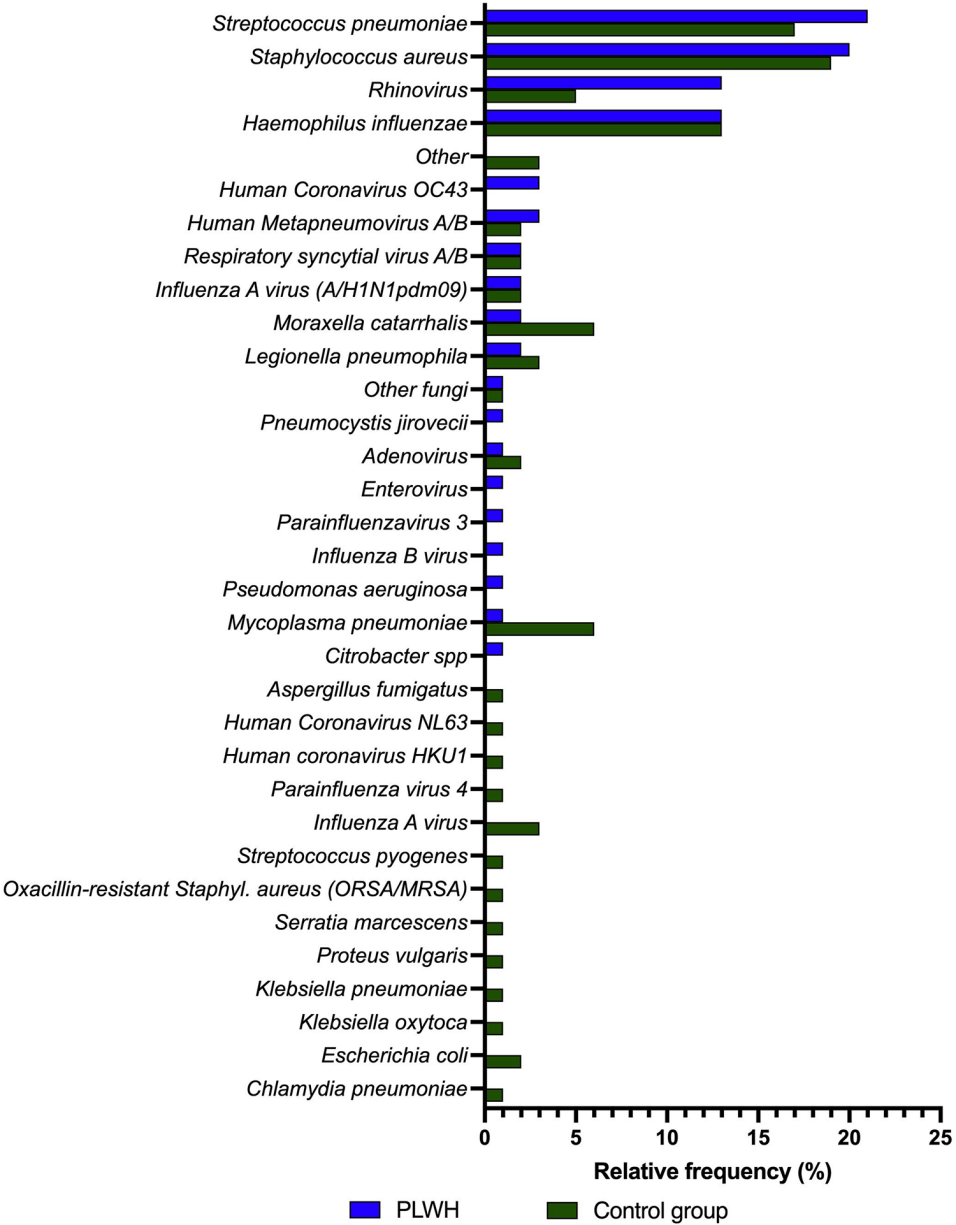
Distribution of pathogens for PLWH and control group. Total number of pathogens: PLWH n = 84, controls: n = 181. Statistical differences between groups were calculated using the Fisher exact test and Bonferroni correction. No significant differences were detected. The following pathogens were grouped together under "other": control group: *Salmonella Serovar Enteritidis* (n = 1), *Streptococcus mitis* (n = 1), Sputum culture: *Arcanobacterium haemolyticum* (n = 1). BAL culture: *Prevotella melaninogenica* (n = 1), *Prevotella denticola* (n = 1), *Capnocytophaga spp.* (n = 1)

Overall, the most frequently identified pathogens were *Streptococcus pneumoniae*, *Staphylococcus aureus, Rhinovirus* and *Haemophilus influenza.* Most frequently detected were *Streptococcus pneumoniae* for PLWH and *Staphylococcus aureus* for controls, and correspondingly vice versa for the second most common pathogen detected. Rhinovirus was detected more frequently in PLWH compared to controls group (PLWH: n = 12 (13.5%), control group: n = 10 (5.1%); n. s.). Remarkably this result showed significance before Bonferroni correction (p = 0.017). Mixed infections with other pathogens and Rhinovirus were observed to be more frequently in PLWH (n = 9 (10.1%) vs. n = 10 (2.5%); p = 0.014). For this analysis each pathogen was counted only once per patient even if it was detected several times in different materials (Fig. 3).

PLWH with evidence of *Staphylococcus aureus* (n = 18) were compared with study patients who tested negative (n = 55). We found no differences of PLWH who tested positive for *Staphylococcus aureus* compared to those without. By comparing PLWH with *Staphylococcus aureus* with those without (HIV positive and negative, n = 235) we found significant differences for BMI (PLWH with *Staph. aureus*: 21.2 (20–23.6) vs. study patients without *Staph. aureus*: 25.5 (22.8–29.75); p < 0.01) and hospital admission (PLWH with *Staph. aureus*: 11/18 (61%) vs. all patients without *Staph. aureus*: 218/235 (93%); p = 0.001). Supplement Table 4 shows the investigated parameters in detail.

For the analysis stratified by material, every pathogen was counted separately: In total, 14 and 49 pathogens were detected by culture from blood, sputum and BAL, for PLWH and controls respectively.

By multiplex-PCR from sputum, nasopharyngeal swab and BAL 106 (PLWH) and 126 (controls) pathogens were detected, respectively. The most common pathogens in both groups were *Streptococcus pneumoniae* (PLWH: 23 (21.7%), control group: 20 (15.9%)) and *Staphylococcus aureus* (PLWH: 20 (18.9%), control group: 31 (24.6%)).

Urine antigen testing revealed a pathogen in 14 cases. Antigen of *Streptococcus pneumoniae* was found in 12 (4 PLWH; 8 Controls) cases and *legionella* in 5 (1 PLWH; 4 Controls) cases. Supplement Tables 5 and 6 show the detected pathogens sorted by diagnostic method for the HIV-positive study group and matched HIV-negative controls. In 7 cases (3 PLWH, 4 controls) with pneumococcal antigen detection in urine, *Streptococcus pneumoniae* was as well detected in culture or PCR. None of the patients tested positive for Legionella antigen had a corresponding bacterial detection in culture or PCR.

### Intended investigation of the pathogen spectrum depending on HIV specific parameters

During the acute CAP illness 65/73 (89%) study patients were on an antiretroviral regimen (ART). In the distribution of the different ART subgroups, nucleoside reverse transcriptase inhibitors (NRTI) were the most common at 60.3% (n = 44/73), integrase inhibitors (INSTI) were the second most common at 37.0% (n = 27/73), and non nucleoside reverse transcriptase inhibitors (NNRTI) and protease inhibitors (PI) were taken by 19.2% (n = 14/73) each.

The subgroup analysis concerning previous and current CD4 and viral load, HIV treatment status, HIV treatment class and CDC-Stadium was not rewarding, because of the small subgroup case numbers (see Supplement Table 7).

## Discussion

To our knowledge, this is the first investigation on pathogen spectrum in PLWH with CAP in more than a decade and the first clinical investigation comparing matched HIV negative controls in a prospective study design.

This investigation’s result confirms the relevance of Pneumococci and *Haemophilus influenzae* as frequently detected infectious agents for CAP in PLWH. The frequent observation of *Staphylococcus aureus* is an unexpected result and a matter of debate since the germ was only rarely detected in previous studies [3, 6]. A limitation of our work is that we cannot distinguish between infection or colonization in our cohort, especially in sputum specimen and nasopharyngeal swab were *Staphylococcus aureus* was predominantly detected by multiplex-PCR. Therefore we conducted a subgroup analysis of PLWH with evidence of *Staphylococcus aureus* versus those patients without. We found a significant result for low BMI reflecting that the evidence of *Staphylococcus aureus* in PLWH with BMI below 21 could be more indicative of an infection, but overall no plausible parameter for the differentiation between *Staphylococcus aureus* infection versus colonization could be identified. Remarkably, *Staphylococcus aureus* was detectable in 6 of 8 of those patients who died and in 5 by PCR from nasopharyngeal swab. Thus the clinical and therapeutic consequences, especially in critically ill patients, still have to be investigated in further studies with a larger number of cases. In the meantime *Staphylococcus aureus* should be taken into account when making empirical therapy decisions. However, there is no evidence of MRSA, so that MSSA-effective empiric therapy is sufficient. Typical HIV- associated pathogens were found only exceptionally (e.g., Pneumocystis: 1, no aspergillus), indicating adequate clinical patient selection to address study scope. This investigation found Pseudomonas only once, i.e., in contrast to prior trials [4, 6], when *Pseudomonas* was linked to low CD4 cell counts and intravenous drug use, but hardly statistically significant. Based on our observation, empirical CAP antibiotic regimen including *Pseudomonas*-active combinations does not seem to be mandatory for PLWH without additional risk, and aminopenicilline-based antibiotics might be sufficient. When in doubt, *Staphylococcus aureus* (MSSA) can be reliably covered by combining it with clavulanic acid.

Although the identification rate of CAP pathogens in PLWH was higher than in large pre-HAART studies [6, 7], pneumococci continued to predominate. Compared with historical data, PLWH are currently more likely to be vaccinated against Pneumococci, are still frequent smokers, are less likely to use intravenous drugs, are older, and pneumonia may result in lower rates of death and bacteremia. Pneumococcal vaccination rates were three times higher in PLWH than in controls, most likely due to better engagement in HIV medical care with regular follow-up. Nevertheless, the incidence of pneumococcal pneumonia was similar in both groups. This underscores the clinical need for effective prevention strategies, i.e., increasing vaccination rates. A limitation of the study was the frequent lack of history on the type of vaccination. Therefore, this analysis cannot measure the benefit of specific vaccination types or strategies. In addition, our study did not collect results of resistance testing. On the other hand, pneumococcal penicillin resistance is unlikely in Germany (0.5%) [8] and is not increased in PLWH [9].

For the first time, multiplex PCR was used for pathogen detection in a cross-sectional study of PLWH with CAP compared with HIV-negative controls. Analogous to another cohort [10] and case descriptions [11–13], rhinoviruses were frequently detected in CAP (third most common infectious agent) and were more frequently observed in PLWH than in controls. A recent retrospective cohort study compared the viral spectrum of pneumonia in 370 (non-HIV) immunocompromised with 436 immunocompetent individuals. Rhinoviruses were found significantly more frequently in the immunocompetent group, along with RSV, adenoviruses, and parainfluenza viruses, during the non-influenza season [14]. Whether rhinoviruses are actually the CAP-pathogen in immunosuppressed patients, or whether they just colonize, can be debated. Interestingly, we observed significantly more mixed infections with rhinovirus and other pathogens in PLWH than in controls. The observation of rhinovirus may be explained by the fact that our cohort was recruited more recently than previous data on pathogen spectrum in PLWH and thus modern PCR based detection methods are available for recruiting CAPNETZ centers.

One of our most striking observations, was a higher mortality for PLWH. All of them were classified CDC disease stage C3. Before 2000, CAP-associated mortality was substantially higher (21 and 27%) [7, 15]. However, statistically, no parameter significantly associated with death could be proven.

Considering patient characteristics, our study population was older than in prior studies [6, 7], and following recommendations for earlier initiation of ART [16], we found higher CD4 cell counts on ART. Following other mega-trends in PLWH population characteristics, the rate of smokers and IV drug users were lower than in historical comparisons [6, 7], and this may impact on both pathogen spectrum and mortality in PLWH and CAP-event. Subsequently, we observed a significant CD4 drop during the CAP event. Overall, this paraclinical observation is not uncommon during the course of infection in the daily routine, but this finding was presented for the first time for CAP in PLWH in a prospective clinical study, although relevant CD4 data were available for only 27, 54, 30, and 34 cases, respectively. Drop of CD4 cells and a corresponding increase of HIV viral load were both observed before in clinical studies with regard to syphilis infection [17–19] and a study on 84 PLWH that was published in 2018 showed a relative non-CD4 specific lymphopenia induced by syphilis infection [20]. In order to be able to better understand the effect of CAP in particular and of infections in general on the course of CD4, this observation should be examined in further studies with a larger number of cases.

A strength of our analysis was the systematic modern diagnostic approach, including atypical CAP-pathogens. Furthermore, we not only investigated respiratory tract specimen for pneumonia, based on culture as conducted by previous studies [6, 7], but in addition, on blood and urine for nucleic acid amplification and antigen testing, in order to explore for CAP-pathogen, in connection with HIV status.

Besides the current investigation data basis is small, compared to the historical trials, this analysis warrants reproduction at high subject numbers without data gaps, for confirmation of CAP-associated increase in HIV-related mortality, and to evaluate the clinical impact of the demonstrated CD4-cell count-drop and exploration of preventive pneumococcus vaccine strategies. Our publication underscores the clinical importance of CAP for PLWH. Affected patients should be treated empirically with a Pneumococci and *Haemophilus influenzae* -effective antibiotic regimen, e.g., Amoxicillin/Clavulanic acid, that covers potential *Staphylococcus aureus* (MSSA) infection as well.

### Supplementary Information

Below is the link to the electronic supplementary material.

Supplementary file1 (PDF 468 KB)

## Data Availability

The data are stored electronically within the CAPNETZ database and are available from CAPNETZ on reasonable request.
