# Supplementary material for: Pathogen spectrum of community acquired pneumonia in people living with HIV (PLWH) in the German CAPNETZ-Cohort
Source: Infection. 2023 Jul 9;52(1):129–37. doi: 10.1007/s15010-023-02070-3 (PMC10811115; doi:10.1007/s15010-023-02070-3)
Supplement: Supplementary file 1 — Supplementary file1 (PDF 468 KB) [file 15010_2023_2070_MOESM1_ESM.pdf]

Including study sites

73 study patients: Basel (n=5), Berlin (3 sites n=1, n=4, n=6), Bonn (n=5), Dresden (n=3), Frankfurt (2 sites n=6, n=1), Hamburg (2 sites n=6, n=14), Jena (n=8), Cologne (2 Sites n=1, n=2), Lübeck (n=1), Maastricht (n=1), Munich (n=8) and Pisa (n=1). 218 control patients: Aachen (n=5), Bad Arolsen (n=8), Basel (n=20), Berlin (4 sites n=2, n=5, n=5, n=20), Bochum (n=1), Bonn (n=4), Cottbus (n=33), Dortmund (n=5), Dresden (n=4), Frankfurt (n=20), Gerlingen (n=4), Hamburg (n=1), Heidelberg (n=1), Jena (n=1), Lübeck (2 sites n=16, n=2), Munich (n=22), Paderborn (n=7), Pisa (n=5), Rotenburg (n=23), St. Gallen (n=1), Wien (n=3).

Supplementary Table 1: CD4 and viral load of PLWH at different time points of the study period:

CD4 median (IQR) at different time points:	
CD4-Nadir (n=43)	167 (44-266)
CD4 absolute (at the time of HIV diagnosis) (n=36)	279 (163.5-611.25)
CD4 absolute (past 3-6 months before CAP) (n=54)	448 (285.75-689.25)
CD4 absolute (during CAP) (n=27)	376 (158-625)
CD4 absolute (28d) (n=20)	561 (192.75-893.75)
CD4 absolute (180d) (n=34)	558.5 (394.25-764.75
HIV-RNA result available at CAP time (n=29/73)	
<50 copies/mL	17/29 (58.6 %)
≥50 copies/mL	12/29 (41.4 %)
HIV-RNA result available at past 3-6 months before CAP (n=53)	
<50 copies/mL	34/53 (64.2 %)
≥50 copies/mL	19/53 (35.8 %)
HIV-RNA result available at day 28 (n=17)	
<50 copies/mL	9/17 (52.9 %)
≥50 copies/mL	8/17 (47.1 %)
HIV-RNA result available at day 180 (n=34)	
<50 copies/mL	27/34 (79.4 %)
≥50 copies/mL	7/34 (20.6 %)
HIV-RNA median (IQR) at different time points:	
HIV viral load (past 3-6 months before CAP) (n=53)	40 (20-79.5)
HIV viral load (during CAP) (n=29)	39 (20-11810)
HIV viral load (28d) (n=17)	49 (23.5-6682.5)
HIV viral load (180d) (n=34)	21 (19-49)

Supplementary Table 2: Comparison of patient characteristics based on HIV viral load and CD4:

	VL < 50 (n=17)	VL ≥ 50 (n=12)	p-value	CD4 <200 (n=9)	CD4 ≥200 (n=18)	p-value
Immune status CD4 [median (Q1-Q3)]	490 (236-677), n=15	278 (10.5-474.5) , n=9	0.222	27 (10.5-158.5), n=9	502 (352.25-683.5), n=18	<,001
Coinfektion with pneumococci	5/17	7/12	0.148	5/9	6/18	0,411
Viral coinfektion	7/17	5/12	>0.999	3/9	9/18	0,683
CDC stage A	5/16	2/10	0.662	0/8	7/17	0,057
CDC stage B	4/16	1/10	0.355	1/8	5/17	0,624
CDC stage C	7/16	7/10	0.704	**7/8**	**5/17**	**0,011**
Time since HIV ED years [median (Q1-Q3)]	19 (4.5-27), n=17	10 (1-16), n=11	0.890	5.5 (1.5-15.25), n=8	16 (5.75-26), n=18	0,066
Time since ART start years [median (Q1-Q3)]	13 (4-21), n=17	4.5 (0-11.25), n=8	0.327	3 (1.0-13. 5), n=5	10.5 (3.75-18.25), n=18	0,263
still ongoing therapy due to OI	0/17	3/12	0.060	3/9	8/18	0,692
anti-infective prophylaxis	3/17	4/12	0.403	3/9	1/18	0,093
age [median (Q1-Q3)]	52 (37.5 - 60) , n=17	41.5 (33.25 - 58.25) , n=12	0.736	46 (37-55), n=9	47 (35.25-56.75), n=18	0,877
BMI [median (Q1-Q3)]	22.75 (21.75 - 24.85) , n=16	20.75 (19.65 - 22.23) , n=10	0.155	**20.1 (18.8-21.3), n=8**	**22.8 (21.3-24.9), n=16**	**0,022**
Vaccination status pneumococci	6/17	2/11	0.419	2/8	7/18	0,667
Vaccination status influenza	5/17	2/11	0.668	0/8	5/18	0,281
male	14/17	12/12	0.246	8/9	16/18	>0.999
COPD	2/17	1/12	>0.999	1/9	1/18	>0.999
smoking never	7/17	2/12	0.234	1/9	7/18	0,201
smoking current	**8/17 (47%)**	**9/12 (75%)**	**0.041**	7/9	9/18	0,231
smoking ex	2/17	1/12	>0.999	1/9	2/18	>0.999
CRB-65 0	8/14	6/10	>0.999	5/8	8/14	>0.999
CRB-65 1	6/14	3/10	0.420	3/8	6/14	>0.999
CRB-65 2	0/14	1/10	0.417	0/8	0/14	>0.999
Inpatient treatment	5/17	4/12	>0.999	**9/9**	**9/18**	**0,012**
ICU	1/17	0/12	>0.999	1/9	1/11	>0.999

Supplementary Table 3: CDC-Stage and pathogens of deceased subjects:

HIV status	Time in years between initial HIV diagnosis and CAP	CDC-Stage	microbiological detected agents (diagnostic method)	
no	-	-	Staphylococcus aureus (NP-Swab-PCR)	
yes	8	C3	Staphylococcus aureus (NP-swab-PCR)	
yes	17	C3	Staphylococcus aureus (NP-swab-PCR)	
yes	27	C3	Staphylococcus aureus (NP-swab-PCR)	
yes	33	C3	Staphylococcus aureus (NP-swab-PCR)	Propionibacterium sp. (blood culture)
no	-	-	Escherichia coli (sputum culture)	Candida albicans (sputum culture)
no	-	-	no pathogen detected	
yes	19	C3	Influenza-B-Virus (NP-swab-PCR)	Staphylococcus aureus (Blood culture, sputum culture sputum, NP-swab-PCR)

Supplementary Table 4: Comparison of patient characteristics based on the presence of Staphylococcus aureus:

	HIV with SA	HIV without SA	p-value (HIV with vs. without SA)	All without SA	p-value (HIV with SA vs. All without SA)
Immune status CD4 [median (Q1-Q3)]	237.5 (28.5 – 432.5). n=8	435.0 (159.0 – 632.0). n=19	0.118		
Coinfection with pneumococci (n/N)	3/18	19/55	0.237	47/235	0.999
viral coinfection	6/18	23/55	0.589	88/235	0.805
CDC stage A	6/18	14/45	0.999		
CDC stage B	3/18	8/45	0.999		
CDC stage C	9/18	21/45	0.999		
Time since initial HIV diagnosis [years] [median (Q1-Q3)]	10.5 (6.5 – 19.5) n=18	12.0 (3.5 – 21.5) n=53	0.948		
Time since initial ART [years] [median (Q1-Q3)]	7 (1.25 - 15.75) n=12	10 (3.5 - 18.0) n=45	0.905		
still ongoing therapy due to OI	4/18	7/55	0.447		
anti-infective prophylaxis	3/18	13/55	0.745		
Age [median (Q1-Q3)]	51 (30 - 60), n=18	53 (39 - 61), n=55	0.823	53 (40 - 62), n=235	0.686
BMI [median (Q1-Q3)]	21.2 (20.0 - 23.6), n=17	23.4 (20.4 - 26.2), n=51	0.200	25.5 (22.8 - 29.75), n=225	<0.01
Vaccination status pneumococci	5/17	15/53	0.999	31/216	0.1525
Vaccination status influenza	7/18	18/53	0.557	50/218	0.1521
Male	17/18	47/55	0.437	206/235	0.7045
COPD	1/18	7/54	0.670	37/232	0.3244
smoking never	5/18	17/ 53	0.999	77/226	0.7962
smoking current	8/18	27/ 53	0.786	79/226	0.4492
smoking ex	5/18	9/ 53	0.324	70/226	0.999

CRB-65 0	11/16	26/46	0.555	132/203	0.999
CRB-65 1	4/16	18/46	0.375	64/203	0.7807
CRB-65 2	1/16	2/46	0.999	7/203	0.4606
Inpatient treatment	11/18	39/55	0.560	218/235	0.0005
ICU	0/11	1/39	0.999	3/218	0.999

Supplementary Table 5: Microbiological agents detected by PCR from respiratory material in the HIV-positive study group (PLWH) and HIV-negative controls:

PLWH				Controls			
**Material**	**Agent**	**N**	**Percent**	**Material**	**Agent**	**N**	**Percent**
**Nasopharyngeal swab multiplex -PCR (n=68)**	Chlamydia pneumoniae	1	1.5 %	**Nasopharyngeal swab multiplex -PCR (n=192)**	Haemophilus influenzae	19	9.9 %
	Haemophilus influenzae	15	22.1 %		Legionella pneumophila	1	0.5 %
	Moraxella catarrhalis	7	10.3 %		Moraxella catarrhalis	6	3.1 %
	Mycoplasma pneumoniae	6	8.8 %		Mycoplasma pneumoniae	14	7.3 %
	Staphylococcus aureus	22	32.4 %		Staphylococcus aureus	31	16.4 %
	Streptococcus pneumoniae	20	29.4 %		Streptococcus pneumoniae	20	10.4 %
	Influenza A virus (A/H1N1pdm09)	1	1.5 %		Influenza A virus	4	2.1 %
	Influenza B virus	1	1.5 %		Influenza A virus (A/H1N1pdm09)	5	2.6 %
	Respiratory syncytial virus A/B	3	4.4 %		Respiratory syncytial virus A/B	3	1.6 %
	Humanes Metapneumovirus A/B	3	4.4 %		Humanes Metapneumovirus A/B	3	1.6 %
	Parainfluenzavirus 4	1	1.5 %		Parainfluenzavirus 3	1	0.5 %
	Humanes Coronavirus HKU1	2	2.9 %		Humanes Coronavirus OC43	1	0.5 %
	Humanes Coronavirus NL63	2	2.9 %		Rhinovirus	8	4.2 %
	Humanes Coronavirus OC43	2	2.9 %		Adenovirus	3	1.6 %
	Rhinovirus	14	20.6 %				
	Enterovirus	1	1.5 %				
	Adenovirus	2	2.9 %				
**Total**		**103**	151.5 %	**Total**		**119**	62.0 %
**BAL PCR (n=6)**	Legionella pneumophila	1	16.7 %	**BAL PCR (n=10)**	Influenza A virus	1	10.0 %
	Staphylococcus aureus	1	16.7 %				
	Pneumocy+stis jirovecii	1	16.7 %				
**TTotal**		**3**	**50.0 %**	**Total**		**1**	**10.0 %**
**Sputum PCR (n=1)**				**Sputum PCR (n=18)**	Chlamydia pneumoniae	1	5.6 %
					Escherichia coli	1	5.6 %
					Mycoplasma pneumoniae	3	16.7 %
					Influenza A virus	1	5.6 %
**Total**		**0**	**0.0 %**	**Total**		**6**	33.3 %

Supplementary Table 6: Microbiological agents detected from cultures of blood and respiratory material in the HIV-positive study group (PLWH) and HIV-negative controls:

PLWH			
**Material**	**Agent**	**N**	**Percent**
Blood culture (n=31)	Staphylococcus aureus	1	3.2 %
	Streptococcus pneumoniae	1	3.2 %
	Cornynebacterium jeikeium	1	3.2 %
	Propionibacterium	1	3.2 %
**Total**		**4**	12.9 %
Sputum culture (n=31)	Citrobacter spp	1	3.2 %
	Haemophilus influenzae	1	3.2 %
	Pseudomonas aeruginosa	1	3.2 %
	Staphylococcus aureus	2	6.5 %
	Streptococcus pneumoniae	2	6.5 %
	Candida spp.	1	3.2 %
	Candida albicans	1	3.2 %
**Total**		**9**	29.0 %
BAL culture (n=10)	Oral / pharyngeal flora	1	10.0 %
**Total**		**1**	10.0 %

Controls			
**Material**	**Agent**	**N**	**Percent**
Blood culture (n=118)	Streptococcus pneumoniae	4	3.4 %
	Streptococcus pyogenes	1	0.8 %
	Cutibacterium acnes	1	0.8 %
	Propionibacterium sp.	1	0.8 %
	Salmonella Serovar Enteritidis	1	0.8 %
	Staphylococcus epidermidis	1	0.8 %
	Staphylococus hominis	2	1.7 %
	Streptococcus mitis	1	0.8 %
	Corynebacterium spezies	1	0.8 %
**Total**		**13**	11.0 %
Sputum culture (n=76)	Escherichia coli	1	1.3 %
	Haemophilus influenzae	3	3.9 %
	Klebsiella oxytoca	1	1.3 %
	Klebsiella pneumoniae	1	1.3 %
	Moraxella catarrhalis	1	1.3 %
	Proteus vulgaris	1	1.3 %
	Serratia marcescens	1	1.3 %
	Staphylococcus aureus	1	1.3 %
	Staphylococcus aureus. Oxacillin-resistant (ORSA/MRSA)	1	1.3 %
	Streptococcus pneumoniae	3	3.9 %
	Streptococcus pyogenes	1	1.3 %
	Candida spp.	2	2.6 %
	Other fungi	3	3.9 %
	Aspergillus fumigatus	1	1.3 %
	Candida albicans	7	9.2 %
	Candida glabrata	1	1.3 %
	Arcanobacterium haemolyticum	1	1.3 %
**Total**		**30**	39.5 %
BAL culture (n=16)	Escherichia coli	1	6.3 %
	Staphylococcus aureus	2	12.5 %
	Prevotella melaninogenica	1	6.3 %
	Prevotella denticola	1	6.3 %
	Capnocytophaga spp.	1	6.3 %
**Total**		**6**	37.5 %

Supplementary Table 7: Subgroup analysis of pathogens concerning previous and current CD4 and viral load. HIV treatment status. HIV treatment class and CDC-Stadium in the HIV-positive study group (PLWH):

microbiological detected agent	CD4				HI viral load				Stay at ICU at the time of CAP		Under ART		CDC-Stage								ART			
	3-6 mo ago		During CAP		3-6 mo ago		During CAP						A			B			C					
	>= 350	< 350	>= 350	< 350	< 50	>= 50	< 50	>= 50	no	yes	yes	no	A1	A2	A3	B1	B2	B3	C2	C3	NRTI	NNRTI	PI	INI
Citrobacter spp	0	1	0	1	1	0	1	0	1	0	1	0	0	0	0	0	0	0	1	0	0	0	1	0
Chlamydia pneumoniae	0	0	0	0	0	0	0	0	2	0	0	0	0	0	0	0	0	0	0	0	0	0	0	0
Escherichia coli	0	0	0	0	0	0	0	0	3	0	0	0	0	0	0	0	0	0	0	0	0	0	0	0
Haemophilus influenzae	4	5	3	0	6	3	2	1	29	2	6	2	2	0	2	0	2	4	0	1	8	3	2	3
Klebsiella oxytoca	0	0	0	0	0	0	0	0	1	0	0	0	0	0	0	0	0	0	0	0	0	0	0	0
Klebsiella pneumoniae	0	0	0	0	0	0	0	0	1	0	0	0	0	0	0	0	0	0	0	0	0	0	0	0
Legionella pneumophila	0	0	1	0	0	0	1	0	7	0	0	0	0	1	0	0	0	0	0	1	2	0	0	1
Moraxella catarrhalis	2	0	0	0	1	1	1	0	13	1	1	0	0	0	0	0	2	0	0	0	2	0	1	0
Mycoplasma pneumoniae	0	1	0	1	1	0	1	0	11	0	1	0	0	0	0	0	1	0	0	0	1	1	0	0
Proteus vulgaris	0	0	0	0	0	0	0	0	1	0	0	0	0	0	0	0	0	0	0	0	0	0	0	0
Pseudomonas aeruginosa	0	1	0	1	1	0	1	0	1	0	1	0	0	0	0	0	0	0	1	0	0	0	1	0
Serratia marcescens	0	0	0	0	0	0	0	0	1	0	0	0	0	0	0	0	0	0	0	0	0	0	0	0
Staphylococcus aureus	4	10	6	2	7	7	3	2	46	2	7	4	4	1	1	0	1	2	1	8	9	3	2	6
Staphylococcus aureus. Oxacillin-resistant (ORSA/MRSA)	0	0	0	0	0	0	0	0	1	0	0	0	0	0	0	0	0	0	0	0	0	0	0	0
Streptococcus pneumoniae	5	9	6	2	9	5	3	6	42	2	9	3	4	3	1	0	0	2	0	5	9	5	4	6
Streptococcus pyogenes	0	0	0	0	0	0	0	0	2	0	0	0	0	0	0	0	0	0	0	0	0	0	0	0
anaerobes	0	0	0	0	0	0	0	0	1	0	0	0	0	0	0	0	0	0	0	0	0	0	0	0
Influenza-A-Virus (universal)	0	0	0	0	0	0	0	0	5	0	0	0	0	0	0	0	0	0	0	0	0	0	0	0
Influenza-A-Virus (A/H1N1pdm09)	1	1	0	1	1	1	0	1	5	0	1	0	0	0	1	0	0	0	0	1	2	0	0	1

Influenza-B-Virus	1	0	1	0	1	0	1	0	1	0	1	0	0	0	0	0	0	0	0	1	1	0	1	0
Respiratory-Syncytial-Virus A/B	0	1	0	1	0	1	0	1	5	0	0	0	1	0	0	1	0	0	0	0	2	0	0	1
Humanes Metapneumovirus A/B	0	1	0	0	1	1	0	1	5	1	1	0	1	0	0	0	0	0	0	2	3	1	1	0
Parainfluenzavirus 3	0	1	0	0	1	0	0	0	0	0	1	0	1	0	0	0	0	0	0	0	0	0	0	1
Parainfluenzavirus 4	0	0	0	0	0	0	0	0	1	0	0	0	0	0	0	0	0	0	0	0	0	0	0	0
Humanes Coronavirus HKU1	0	0	0	0	0	0	0	0	2	0	0	0	0	0	0	0	0	0	0	0	0	0	0	0
Humanes Coronavirus NL63	0	0	0	0	0	0	0	0	2	0	0	0	0	0	0	0	0	0	0	0	0	0	0	0
Humanes Coronavirus OC43	0	2	0	0	1	1	0	0	1	0	1	0	2	0	0	0	0	0	0	0	2	1	0	0
Rhinovirus	5	7	4	3	5	6	4	2	16	1	5	1	1	2	0	0	1	0	0	6	6	2	3	5
Enterovirus	0	0	0	0	0	0	0	0	0	0	0	0	1	0	0	0	0	0	0	0	1	0	0	0
Adenovirus	0	1	0	1	1	0	1	0	4	0	1	0	1	0	0	0	0	0	0	0	1	0	0	1
Pneumocystis jirovecii	0	0	0	0	0	0	0	1	1	0	0	0	0	0	0	0	0	0	0	1	1	0	0	1
Candida spp.	0	1	0	0	0	1	0	0	2	0	0	0	1	0	0	0	0	0	0	0	1	0	0	0
Other fungi	0	0	1	0	0	0	0	1	3	0	0	1	0	0	0	0	0	0	0	0	0	0	0	0
Aspergillus fumigatus	0	0	0	0	0	0	0	0	1	0	0	0	0	0	0	0	0	0	0	0	0	0	0	0
Candida albicans	1	0	1	0	0	1	1	0	8	0	0	0	0	0	0	0	0	0	0	1	1	0	1	0
Candida glabrata	0	0	0	0	0	0	0	0	1	0	0	0	0	0	0	0	0	0	0	0	0	0	0	0
Other	0	1	1	1	1	0	1	1	15	0	1	0	0	0	0	1	0	0	0	1	3	0	0	1
**Total**	**12**	**24**	**12**	**9**	**22**	**14**	**11**	**11**	**151**	**5**	**22**	**7**	**8**	**5**	**3**	**1**	**4**	**4**	**2**	**19**	**30**	**9**	**10**	**17**

Supplementary Table 8: Frequency of diagnostics for pathogen detection in the HIV-positive study group (PLWH) and HIV-negative controls:

Diagnostics	PLWH (n=73)	Control (n=218)	p-wert (Fisher´s exact test)
BAL culture	16 (20.3)	10 (4.7)	<0.001
Blood culture	31 (42.5)	118 (54.1)	0.104
Sputum culture	31 (42.5)	76 (34.9)	0.264
NP-swab PCR	68 (93.2)	192 (88.1)	0.277
Sputum PCR	1 (1.4)	18 (8.3)	0.052
BAL PCR	6 (8.2)	10 (4.6)	0.244
